# Advertisements for 1878

**Published:** 1877-12-20

**Authors:** 


ADVERTISEMENTS FOR 1878.—The superior advantages of our journal as an advertising medium are now generally understood. The following first-class establishments have already engaged space for the forthcoming volume, to-wit: Messrs Aloe & Hernstein, St. Louis; Billings, Clapp & Co, Boston; Bellevue Hospital Medical College, N. Y.; Bullock & Crenshaw, Philadelphia; Ludden & Bates, Savannah; Merrell, Thorp & Lloyd, Cincinnati; Pemberton, Samuels & Reynolds, Atlanta; Reed & Carnrick, New York; Wm. R. Warner & Co., Wyeth & Bro., Philadelphia.

